# The Story of the Insane from Year to Year

**Published:** 1899-01-07

**Authors:** 


### THE STORY OF THE INSANE FROM YEAR TO YEAR.

(ELEVENTH SERIES.)

### LANARK DISTRICT ASYLUM AT HARTWOOD.

THE average number resident during the year was 468. There were 132 first admissions, 56 readmissions, 85 recoveries, 31 discharged relieved, and 30 deaths. Ten of the deaths were caused by cerebral and spinal diseases, 1 from phthisis, and 3 from pneumonia. Religion is given as the cause of insanity in 10 of the year's admissions, intemperance in 36, hereditary influence in 6, injury to head in 3, and in 50 cases the cause was not ascertained. The recovery rate stands at 45·2 per cent. of the admissions, and the death-rate at 4·7 of the average number resident. The former is considerably above the public asylum average, and the latter much below it. Dr. Campbell Clark is quite right when he says that the medical spirit has undergone much development lately, and that there has been an increasing facility in accepting the idea that insanity is a disease and requires hospital treatment like any other bodily disease. Of course, English physicians have accepted this for many generations, but there are still vast numbers of the laity to be converted to the "hospital" idea of asylums, and we do not think that Dr. Clark need fear that this idea will be pushed too far for many a long year to come. The Visiting Committee say that the asylum has been managed during the year in the same satisfactory manner as before. The weekly charge to the unions is 9s. per head per week. Out-district patients pay 14s.

### SOUTH YORKSHIRE ASYLUM, SHEFFIELD.

ON the first day of January, 1897, there were in this asylum 1,598 patients, and by the end of the year 1,630 were in residence. During the year there were 405 first admissions, 76 readmissions, 184 recoveries, 33 discharged relieved, and 207 deaths. These numbers show that the percentage of recoveries, calculated on the admissions, was 38 2, and that of the deaths, calculated on the average number resident, was 12·8. The former is about the usual county asylum average, and the latter is somewhat above it. Dr. Kay says the death-rate is high owing to the number of deaths from phthisis, pneumonia, and other lung diseases; but he does not say what was the cause of these diseases being so prevalent, and he states that the sanitary state of the asylum is good. Table V. tells us that 22 of the deaths were caused by phthisis, 33 by lung congestion, 20 by pneumonia, 3 by enteritis, and 70 by cerebral and spinal diseases. The usual causes of insanity are of course represented in the year's admissions. Thus 267 of the cases are said to owe their disease to domestic trouble and mental anxiety, 29 to religion, 8 to love, 67 to intemperance, and 128 to hereditary influences. The assistant medical officers have continued their lectures and instructions to the staff, and ten of the nurses have obtained the Medico-Psychological Nursing certificate. This is very good so far as it goes, and we venture to hope that Dr. Kay and his able assistants will not stop here, but that they will enlarge their courses of lectures, and, after a three years' system, that they will themselves examine and certify to their nurses' skill, as is done at the Northampton and Dorset County, and probably other asylums. It is only thus that mental nurses can be brought in line, or perhaps ahead of their hospital sisters. Dr. Kay very wisely gives prominence to outdoor employment in the treatment of his cases, and he thinks that the possession of more land would afford further scope in this direction. Certainly it might do this, but we doubt if it would. More land is very apt to mean more agricultural machinery, and not spade labour. The committee report that "Dr. Kay and the other medical officers have given every care and attention to the patients." The weekly cost of maintenance was 9s. 3d. per head.

### THE LAWN HOSPITAL FOR THE INSANE, LINCOLN.

In this institution there are about 80 patients, and these pay from 30s. a week upwards. Some are admitted at lower rates than 30s. provided the special circumstances of the cases warrant the reduction. The lawn is one of the lunatic hospitals in which making money does not seem to be a paramount object of the committee, and hence it is deserving of praise for what it has done, and is doing, for those insane persons who are unable to afford the higher rates charged in private asylums and in some other hospitals. It is a pity that the number of beds could not be doubled, and so enable the managers to increase the number of deserving cases and reduce the charges. During the year 1897 there were 33 admissions, 13 recoveries, and 4 deaths. The recoveries would stand at 44 per cent. on the admissions, which is a high percentage, and the deaths at 5 per cent. on the average number resident, which is a low one; but, as we have previously said when speaking of small asylums, it is impossible to draw any reliable inference from these percentages. The deaths were all from natural causes, and there was neither restraint nor seclusion used during the year. A party of patients spent a fortnight at the seaside, and others have had occasional leave of absence. Dr. Russell has now an assistant medical officer, and we are glad to note that a lady medical, Miss Lilian Cunard-Cummins, holds the office. The committee "cordially recognise" Dr. Russell's services to the institution.

